# Supplementary material for: Crosstalk between AML and stromal cells triggers acetate secretion through the metabolic rewiring of stromal cells
Source: eLife. 2022 Sep 2;11:e75908. doi: 10.7554/eLife.75908 (PMC9477493; doi:10.7554/eLife.75908)
Supplement: Supplementary file 1. — Table shows information regarding the type of AML, karyotype and additional mutation and risk of the different AML patient samples used during this study. [file elife-75908-supp1.docx]

**Supplementary File 1. Primary AML samples’ additional information**

| **Samples obtained from Martini Hospital** | | | | | |
| --- | --- | --- | --- | --- | --- |
| Patient | Tissue of origin | Type of AML (FAB) | Karyotype | Other mutations | Risk |
| AML1 | PB | M5 | Normal | FLT3-ITD; NPM1; DNMTA3; IDH2 | Adv |
| AML2 | PB | M1 | Unknown | - | Adv |

| **Samples obtained from University Hospital Birmingham NHS Foundation Trust, UoB** | | | | |
| --- | --- | --- | --- | --- |
| Patient | Tissue of origin | Type of disease | Karyotype | Other mutations |
| AML3 | PB | Secondary AML from MDS | 46,XY | TET2; SRSF2;  RUNX1; CEBPA;  ASXL1 |
| AML4 | PB | Secondary AML from MDS | 46,XY | NPM1 neg  FLT3 neg |
| AML5 | PB | AML M1 (FAB) | 46 XX | FLT3-ITD, 2xWT1, CEBPA |
| AML6 | PB | AML M2 (FAB) | 46 XX | - |
| AML7 | PB | AML M4 (FAB) | 46 XY | - |
